# Preventive Effects of Different Fermentation Times of Shuidouchi on Diphenoxylate-Induced Constipation in Mice

**DOI:** 10.3390/foods8030086

**Published:** 2019-03-01

**Authors:** Lianhong Chen, Jing Zhang, Huayi Suo, Wei Wang, Hongwei Wang, Yu Zhang, Qiang Hu, Xin Zhao, Jian Li

**Affiliations:** 1 College of Life Science and Technology, Southwest Minzu University, Chengdu 610041, China; lianhong_chen@163.com; 2 Key Laboratory of Qinghai-Tibetan Plateau Animal Genetic Resource Reservation and Utilization, Southwest Minzu University, Chengdu 610041, China; 3 Chongqing Collaborative Innovation Center for Functional Food, Chongqing University of Education, Chongqing 400067, China; zjinger0810@126.com (J.Z.); zhaoxin@cque.edu.cn (X.Z.); 4 Department of Environmental and Quality Inspection, Chongqing Chemical Industry Vocational College, Chongqing 402160, China; 5 College of Food Science, Southwest University, Chongqing 400715, China; birget@swu.edu.cn (H.S.); wanghw_1978@swu.edu.cn (H.W.); zhangyu_512@sina.cn (Y.Z.); 6 Academy of Animal Sciences and veterinary Medicine, Qinghai University, Xining 810016, China; zhanghui@qh.e-chinalife.com; 7 Bamboo Diseases and Pests Control and Resources Development Key Laboratory of Sichuan Province, Leshan 614000, China; huqiang19834@163.com

**Keywords:** Shuidouchi, fermentation, diphenoxylate, constipation, mRNA

## Abstract

This study compares the prevention effects of Shuidouchi with different fermentation times on constipation in mice. Shuidouchi is a short-time fermented soybean product. By improving its processing technology, it can incur better biological activity and become a health food. The Shuidouchi-treated mice were evaluated using constipation-related kits, quantitative polymerase chain reaction (qPCR), and Western blot assays. After the mice were fed 72-h-fermented Shuidouchi (72-SDC) for 9 d, the defecation time to excrete the first black stool was lower than that of the control and 24-SDC and 48-SDC groups, but was much higher than that of the normal group. The gastrointestinal (GI) transit of the small intestine of the 72-SDC group was higher than that of the control and the 24-SDC and 48-SDC groups, but lower that of the normal group. Meanwhile, 72-SDC could significantly increase the levels of *ghrelin*, endothelin-1 (ET-1), vasoactive intestinal peptide (VIP), and acetylcholinesterase (AchE) in the serum of constipated mice compared to the levels in mice in the control group. Moreover, 72-SDC could raise *c-Kit*, stem cell factor (*SCF*), glial cell-derived neurotrophic factor (*GNDF*), neuronal nitric oxide synthase (*nNOS*), and endothelial nitric oxide synthase (*eNOS*) messenger RNA (mRNA) and protein expression levels, and reduce transient receptor potential cation channel subfamily V member 1 (*TRPV1*) and inducible nitric oxide synthase (*iNOS*) expression levels in small-intestinal tissue compared to the levels in the control group. Meanwhile, 72-SDC also raised *ghrelin* mRNA expression in gastric tissue and transient receptor potential ankyrin 1 (*TRPA1*) mRNA expression in colon tissue compared to the control group mice; these effects were stronger than those of 24-SDC and 48-SDC. Shuidouchi has good preventative effects on constipation and performs best when fermented for at least 72 h.

## 1. Introduction

Douchi is a kind of traditional fermented-soybean product in China, which can be used as a seasoning and as medicine. After long-term consumption, it can promote appetite, eliminate stasis, and dispel wind and dampness. Douchi is a long-time fermented soybean food (ten months), while Shuidouchi is also a fermented soybean food like Douchi; however, the fermentation time is shorter at only three days [1]. Shuidouchi is bacteria-fermented Natto, a kind of brewed flavor food produced by fermenting soybeans with bacteria. The product is in a wet state; thus, its water content is high. During the stacking fermentation process, hydrolysis causes changes in composition [2]. After fermentation, the content of active soybean isoflavones in Shuidouchi increases, making it more convenient for the human body to absorb [3]. Shuidouchi is rich in nutrition, containing proteins, vitamins, and minerals [2,3].

After soaking, soybeans are cooked and fermented at the proper temperature for 2–3 days. When there are viscin threads produced between soybeans, fermentation is complete. Then, auxiliary materials, such as salt, pepper powder, Chinese prickly ash powder, and vegetable oils, are added to the fermented soybeans. To get a stronger taste, the soybeans are fermented under low temperature for one to two weeks [4]. Fermentation time is one of the main factors affecting the quality and nutrient composition of fermented food. If the nutrient composition of the food changes, its effect will change. Traditional Chinese medicine believes that natto can relieve the exterior, clear heat, and remove toxins. The production techniques of natto and Shuidouchi are close, and there were studies in Japan showing that natto has the physiological function to protect the gastrointestinal tract [5].

In modern society, constipation is a common physiological state [6]. The defecation times of people with constipation decrease and, due to the low water content of feces, the number of single defecations decreases, and defecation becomes difficult [7]. Because of the influence of working conditions, approximately 70% of people in modern life are in a subhealth state and have gastrointestinal discomfort, and most of these people have constipation [6]. Food, including health food that can improve health and function, can help people with a suboptimal health status return to normal. Using food to improve constipation is currently advocated as the most important way of improving intestinal health [8]. Soybean products were proven to have a good effect on inhibiting constipation [9], and fermented soybeans were shown to have a better effect on the intestinal tract [10].

Raffinose and genistein are important active ingredients in soybeans [11]. During fermentation, their contents change with the degree of fermentation [12]. Raffinose is an excellent nutritional source and effective multiplication factor of bifidobacteria, *Lactobacillus acidophilus*, and other beneficial bacteria in the human intestinal tract [13]. Cottonseed sugar affects the whole intestine and improves defecation. It can improve the digestive function of the human body, promote the absorption of calcium into the human body, enhance the immunity of the human body, and have obvious effects on the prevention of disease and aging. Raffinose can be used as a main ingredient for protecting the human body and living animal organs, as well as prolonging the survival period of live bacteria [14]. Genistein, which is estrogen-like and has antioxidant activity, can inhibit the activity of protein tyrosine kinase (PTK) and topological isoenzyme II, and can also inhibit angiogenesis [15].

The compound diphenoxylate is a kind of drug used to treat functional diarrhea; it can block receptors on intestinal mucosa and reduce intestinal peristalsis to delay intestinal contents; however, it can lead to constipation under normal conditions [16]. According to this pharmacological function, diphenoxylate can be used to induce constipation in mice to establish an animal constipation model, which became an important experimental method to examine the effects of food on inhibiting constipation [17]. As a common fermented food in China, Shuidouchi is similar to Japanese natto. In Japan, natto was fully studied and improved to obtain high-value-added products [2]. However, Shuidouchi remains to be fully developed and utilized in China [3]. This study will be conducive to the development and utilization of Shuidouchi. By using compound diphenoxylate tablets to induce constipation in mice, this study observes the preventative effects of Shuidouchi with different fermentation times on diphenoxylate-induced constipation to provide new theoretical support for the standardized production of Shuidouchi, as well as basic experimental data for developing Shuidouchi into gastrointestinally functional food. 

## 2. Materials and Methods

### 2.1. Shuidouchi Fermentation

Soybeans were soaked for 12 h in distilled water (two times the weight of the soybeans). The soaked soybeans were placed in a high-pressure steam sterilizer for cooking (60 min, 105 °C). After cooking, the soybeans were cooled to 45 °C; the steamed bean water was filtered and kept in cold storage. The cooled soybeans were fermented in a constant temperature incubator at 40 °C for 24, 48, and 72 h, respectively. Lastly, the steam bean water (10% of the soybean weight) was added to the fermented soybean to make the Shuidouchi [2].

### 2.2. Determination of pH and Acidity

Samples of Shuidouchi were diluted 1:10 with distilled water. Then, the pH value of Shuidouchi was determined as follows: 1 g of Shuidouchi was diluted with 20 mL of distilled water and titrated to pH = 8.3 by 0.1 mol/L NaOH, and the acidity of the sample was calculated by the following formula: acidity (%) = amount of NaOH used for titration × dilution multiplier × 0.09 × 100/sample weight (g) [2].

### 2.3. Determination of Total Number of Bacteria

Firstly, 1 g of Shuidouchi or 0.1 g of feces was diluted 1:10 with sterilized physiological saline; then, the sample solution was filtered. The number of colonies was observed at 30 °C on a 9-cm plate count agar (tryptone 5.0 g, yeast extract 2.5 g, glucose 1.0 g, agar 15.0 g dissolved in 1000 mL of distilled water, Shanghai Yuanmu Biotechnology Co., Ltd., Shanghai, China) plate at 30 °C, and the approximate total number of bacteria (colony-forming units (CFU)/g) was calculated [3].

### 2.4. Isolation and Identification of Microorganisms

Firstly, 90 mL of sterile saline was added to 10 g of Shuidouchi for grinding and stirring, and the supernatant was removed and diluted by a factor of 10^−7^. The diluent was coated on the Luria–Bertani solid medium and cultured at 28 °C for 48 h. The dominant colonies were observed by the naked eye and cultured in Luria–Bertani liquid medium. Then, the obtained bacteria were identified by physiological and biochemical experiments. Then, the isolated bacteria were sequenced by 16s RNA, the primers were 27F (AGAGTTTGATCCTGGCTCAG) and 1492R (TACGYTACCTTGTTACGACT), and the PCR products were sequenced. The sequenced bacteria were compared and analyzed by the basic local alignment search tool (BLAST) program of National Center for Biotechnology Information (NCBI).

### 2.5. Determination of Raffinose Content

A 1-g raffinose standard was placed in a 10-mL volumetric flask and fixed with 80% ethanol (*v*/*v*). The solution was diluted with 80% ethanol and diluted to 20, 40, 60, 80, and 100 mg/mL raffinose standard solution. Then, 0.2 mL of the standard solution at different concentrations was taken, 0.1 mL of phenol solution at a concentration of 50 mg/mL was added, 5 mL of concentrated sulfuric acid was added, and the absorbance was determined at 490 nm after heating for 3 min in a 100 °C water bath; the standard curve was drawn through the absorbance and the content of raffinose. The ethanol extract of fermented soya bean was placed in a 10-mL bottle with mixed expansion agent (*v*/*v*/*v*, acetonitrile: glacial acetic acid: water = 6:3:2), and 5 μL of ethanol extract was placed on the thin plate (110 °C activation for 1 h) to make a strip of 3 cm. The thin plate was placed in the cylinder to expand (25 °C constant temperature for 6 h), and the color developer (aniline-diphenylamine-phosphoric acid) was sprayed with color (85 °C for 15 min). The strip area of 1.5 cm × 4 cm was collected in 3 mL of 80% ethanol (*v*/*v*) and was centrifuged at 4500 rpm for 20 min by ultrasonic extraction. Then, the absorbance value was determined by the method of measuring the standard curve of the supernatant to repeat the raffinose standard curve, and the content of raffinose was calculated [18,19].

### 2.6. Determination of Genistein Content

A 5-mg standard for genistein was put in a 25-mL volumetric flask with 80% ethanol (*v*/*v*). The solution was diluted with 80% ethanol and diluted to 1, 2, 3, 4, and 5 g/mL. The absorbance of the standard solution was measured at 271 nm, and the standard curve was drawn by absorbance and genistein content. The content of genistein in the ethanol extract of Shuidouchi was obtained by determining the ethanol extract of fermented soya bean with 1 g in a 100-mL bottle with 80% ethanol and determining the absorbance control standard curve at 271 nm [20,21].

### 2.7. In Vitro Small-Intestine Movement Effect

The five ICR female mice (6 weeks) were used for this experiment. After fasting 24 h, the mice were executed, and the small intestine of the mice was removed and divided into 3 cm long segments. The isolated intestinal segments were moved to the 4 °C magnus’ bath and connected with the muscle tension sensor (JH-2, Beijing Aerospace Engineering Research Institute, Beijing, China) and biomechanical test system (BL-420F, Chengdu Taimeng Technology Co., Ltd., Chengdu, Sichuan, China). The test parameters were set as voltage 20 mV, current frequency 20 Hz, and paper speed 5.00 s/div. The movement effects of the intestine segments were observed, the initial physiological condition (contraction frequency and contractility) of intestinal motility stimulated by 1 mL raffinose (10%, *w*/*v*), genistein (10%, *w*/*v*), 24-SDC (10%, *w*/*v*), 48-SDC (10%, *w*/*v*) and 72-SDC (10%, *w*/*v*) were determined [22].

### 2.8. Induction of Constipation in Mice

**Figure 1:**
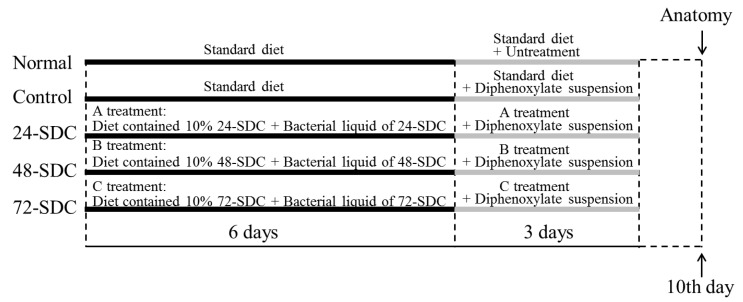
Schematic of the experimental procedure. 24-SDC: 24-h-fermented Shuidouchi; 48-SDC: 48-h-fermented Shuidouchi; 72-SDC: 72-h-fermented Shuidouchi.

The experimental Institute of Cancer Research (ICR) female mice (six weeks) were randomly divided into the normal group, control group, 24-h-fermented Shuidouchi (24-SDC) group, 48-h-fermented Shuidouchi (48-SDC) group, and 72-h-fermented Shuidouchi (72-SDC) group, 10 in each group. The normal group was not treated during the whole experiment. The control group was not treated for the first six days. After six days, the mice were given a diphenoxylate suspension (5 mg/kg) by lavage once a day for three days. These two groups were given free drinking water and normal mouse feed during the experiment. The 24-SDC, 48-SDC, and 72-SDC groups were given a diet containing 10% freeze-dried Shuidouchi with different fermentation times throughout the experimental process, and the mice were given free drinking water at the same time. At the same time, according to the weight of freeze-dried Shuidouchi consumed by mice every day, the corresponding amount of Shuidouchi was crushed and added to 10 times the amount of distilled water (*w*/*v*). After mixing and filtering, the filtrate was centrifuged and separated at 4500 rpm, the upper layer of the filtrate was discarded, and 0.2 mL of distilled water was added. The 0.2 mL solution containing bacteria was given to mice by gastric lavage. On the last three days, the mice were given a diphenoxylate suspension (5 mg/kg) by lavage once a day. After nine days, all the mice fasted for 24 h and were then given 0.1 mL/10 g ice water containing 10% activated carbon by lavage (Figure 1). Each group was divided into two parts. Five mice were used to observe discharge time of the first black stool, while another five mice were given activated carbon ice water for 30 min and were then killed by cervical dislocation to observe the gastrointestinal (GI) transit of activated carbon in the small intestine. The activated carbon propulsive rate was calculated according to the following formula: activated carbon propulsive rate (%) = (propulsion distance of activated carbon in the small intestine (cm)/total length of the small intestine (cm)) × 100 [23]. 

The study was approved (No. SCXK (Yu) 2017-0001) by the Animal Ethics Committee of Chongqing University of Education (Chongqing, China).

### 2.9. Determination of Ghrelin, ET-1, VIP, and AchE Serum Levels

*Ghrelin* regulates the central nervous system, ET-1 regulates cardiovascular function, VIP dilates blood vessels, and AchE regulates bowel contraction. They are markers of constipation. After collection of plasma, the plasma was centrifuged at 4000 rpm for 15 min; then, the supernatant was collected and made into the serum. The serum levels of *ghrelin* (R&D, Minneapolis, MN, USA), ET-1, VIP, and AchE were determined using the respective kits (Nanjing Jiancheng Bioengineering Institute, Nanjing, Jiangsu, China).

### 2.10. Small-Intestine Tissue Hematoxylin and Eosin (H&E) Staining of Sections

Part of the small-intestine tissue was immediately soaked in fresh 10% formalin fixative for H&E-stained section production. The changes in cell morphology in the whole field in the small-intestine tissue samples were observed under a microscope (BX43F, Olympus, Tokyo, Japan) [20].

### 2.11. Quantitative PCR (qPCR) Assay

The gastric tissue and part of the small-intestine tissues of mice were collected and washed using normal saline. The total RNA of small-intestine tissues was extracted by TRIzol reagent. Briefly, 1 μg of extracted RNA was mixed with the mixed reagent (1 μL of oligodT_18_, 1 μL of RNase, 1 μL of deoxy-ribonucleoside triphosphate (dNTP), 1 μL of moloney murine leukemia virus (M-MLV) enzymes, and 10 μL of 5× buffer, Thermo Fisher Scientific, New York, NY, USA) to synthesize complementary (cDNA) under the conditions of 37 °C for 120 min, 99 °C for 4 min, and 4 °C for 3 min. Then, 2 μL of the synthesized cDNA was mixed with 2 μL of total primer (10 μmol/L, Table 1, Thermo Fisher Scientific), 10 μL of 2× SYBR Premix Ex Taq II, 0.4 μL of 50× ROX reference Dye, and 5.6 μL of double-distilled water (ddH_2_O; Thermo Fisher Scientific). Messenger RNA (mRNA) levels were determined using the automatic thermocycler (QuantStudio^TM^ 6 Flex PCR, Life Technologies, Gaithersburg, MD, USA) for 40 cycles at 94 °C for 30 s, 58 °C for 30 s, and 72 °C for 50 s, followed by 10 min at 75 °C. The relative transcription levels of mRNA were calculated using the 2^−ΔΔCr^ formula [24].

### 2.12. Western Blot Assay

The protein in the part of the small-intestine tissues in mice was extracted using a kit (Thermo Fisher Scientific), and the protein concentration was adjusted to 30 µg/mL. Sodium dodecyl phosphate–polyacrylamide gel electrophoresis was conducted using a 10% separation gel and a 5% stacking gel. The protein extracted from the small-intestine tissues was isolated for 2 h using a 5% nonfat milk blocking liquid. The protein was then incubated at 25 °C for 2 h using the primary antibodies of *c-Kit* (14-1172-82, 1:1000 dilution, Thermo Fisher Scientific), stem-cell factor (*SCF*; PA5-20746, 1:1000 dilution, Thermo Fisher Scientific), transient receptor potential cation channel subfamily V member 1 (*TRPV1*; PA5-77317, 1:200 dilution, Thermo Fisher Scientific), glial cell-derived neurotrophic factor (*GDNF*; PA5-77537, 1:1000 dilution, Thermo Fisher Scientific), neuronal nitric oxide synthase (*nNOS*; 37-2800, 1:1000 dilution, Thermo Fisher Scientific), endothelial nitric oxide synthase (*eNOS*; PA1-037, 1:1000 dilution, Thermo Fisher Scientific), inducible nitric oxide synthase (*iNOS*; 14-5920-82, 1:1000 dilution, Thermo Fisher Scientific), and β-actin (MA1-140, 1:5000 dilution, Thermo Fisher Scientific). After treatment with the primary antibody, the sample membrane was soaked in the secondary antibody (A32723, 1:5000 dilution, Thermo Fisher Scientific) solution at 25 °C for 1 h. After washing the membrane 3 times using Tris-buffered saline with Tween (TBST), the gel image system (GIS) gel image was used to photograph the system (iBright™ FL1000 Imaging System, Thermo Fisher Scientific) [24].

### 2.13. Statistical Analysis

Parallel experiments were done three times for each mouse, and the results of the three experiments were averaged; the experimental data were displayed as means ± standard deviation. Differences between the mean values under the level of *p* < 0.05 for each group were assessed by one-way analysis followed by using Tukey’s test for multiple comparisons. Significant differences between either group and the other groups were analyzed. The SAS v9.1 statistical software package (SAS Institute Inc., Cary, NC, USA) was used for the analysis.

## 3. Results

### 3.1. The pH, Acidity, and Total Bacterial Count of Shuidouchi

The physicochemical indexes of Shuidouchi are the basic indicators for judging its quality [2]. As shown in Table 2, 72-SDC had the lowest pH value and the highest acidity and total viable counts. The acidity and total viable counts of 48-SDC were also higher than those of 24-SDC, but the pH value of 24-SDC was the highest.

### 3.2. Identification of Strain from Shuidouchi

The colony of a strain isolated from Shuidouchi was subcircular, milky white, with a folded surface, slightly leafy teeth on the edge, and opaque. Physiological and biochemical tests (Table 3) also showed that the strain isolated from Shuidouchi was similar to that of *Bacillus subtilis*, and was initially considered to belong to the genus *Bacillus*. The BLAST program showed that the bacteria had 99% homology with known *Bacillus subtilis* natto in Gene Bank database.

### 3.3. Raffinose Content in Shuidouchi 

**Figure 2:**
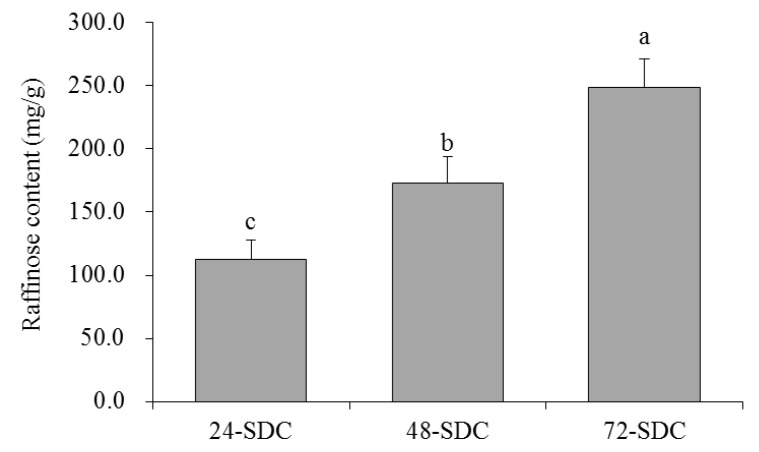
Raffinose content of Shuidouchi at different fermentation times. Values presented are means ± standard deviation (*n* = 3). Different letters indicate significant differences (*p* < 0.05) between each group, and the same letters indicate that there is no significant difference (*p* > 0.05) between each group according to Tukey’s test for multiple comparisons. 24-SDC: 24-h-fermented Shuidouchi; 48-SDC: 48-h-fermented Shuidouchi; 72-SDC: 72-h-fermented Shuidouchi.

Soybean isoflavones are important functional ingredients in Shuidouchi, and the detection of soybean isoflavone is helpful to judge the function of Shuidouchi [25]. The standard curve obtained from the determination of the raffinose standard product was *Y* = 89.804*X* + 0.7893 (*R*^2^ = 0.9975). Through the determination and calculation of samples, the contents of 24-SDC, 48-SDC, and 72-SDC were found to be 112.31, 172.72, and 248.34 mg/g, respectively (Figure 2), whereby 72-SDC had the highest raffinose content.

### 3.4. Genistein Content in Shuidouchi

**Figure 3:**
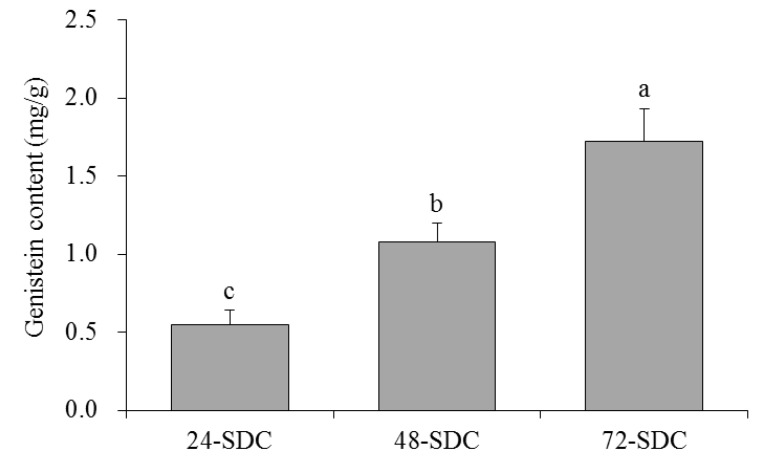
Genistein content of Shuidouchi at different fermentation times. Values presented are means ± standard deviation (*n* = 3). Different letters indicate significant differences (*p* < 0.05) between each group, and the same letters indicate that there is no significant difference (*p* > 0.05) between each group according to Tukey’s test for multiple comparisons. 24-SDC: 24-h-fermented Shuidouchi; 48-SDC: 48-h-fermented Shuidouchi; 72-SDC: 72-h-fermented Shuidouchi.

Genistein is also a soybean isoflavone. The standard curve of genistein content was *Y* = 9.5393*X* + 0.0311 (*R*^2^ = 0.9997), where it was found that 72-SDC had a higher genistein content (1.72 mg/g) than 24-SDC (0.55 mg/g) and 48-SDC (1.08 mg/g) (Figure 3). The longer the fermentation time was, the higher the genistein content was. 

### 3.5. In Vitro Small-Intestine Movement Effect of SDC

Determination of in vitro small-intestine movement effect can preliminarily judge the ability of samples to stimulate intestinal vitality. As shown in Table 4, the genistein solution treatment showed the highest contraction frequency, and raffinose and 72-SDC solutions also showed higher contraction frequencies than those of 24-SDC and 48-SDC solutions. Meanwhile, genistein, raffinose, and 72-SDC solutions showed similar intestinal muscle contractilities; they were significantly (*p* < 0.05) higher than 24-SDC and 48-SDC solutions. These results showed that the in vitro small-intestine movement effect of 72-SDC was close to that of raffinose and genistein.

### 3.6. Stool Status of Shuidouchi-Treated Mice

The determination of fecal state can preliminarily judge the degree of constipation. After inducing constipation, the stool weight, particle counts of stool, water content of stool, and total viable counts of stool of mice (control group) decreased (Table 5). Shuidouchi could inhibit the reduction of stool weight, particle counts of stool, water content of stool, and total viable counts of stool, and the stool status of 72-SDC-treated mice was closest to that of the mice in the normal group.

### 3.7. First Black Stool Defecation Time of Mice

**Figure 4:**
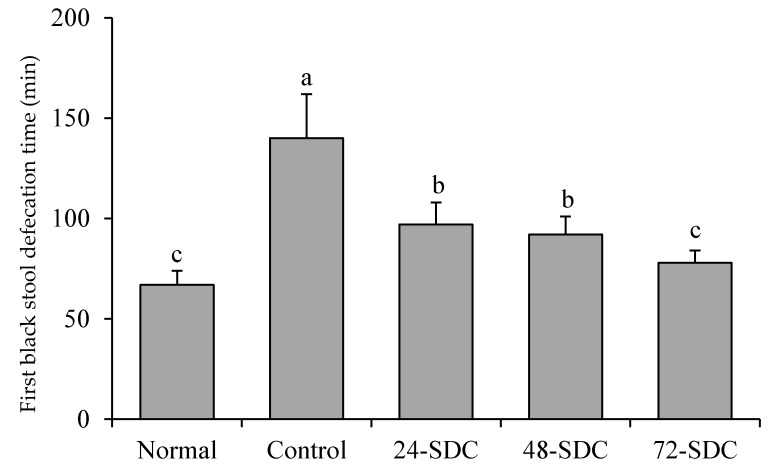
First black stool defecation time of mice after diphenoxylate treatment. Values presented are means ± standard deviation (*n* = 5). Different letters indicate significant differences (*p* < 0.05) between each group, and the same letters indicate that there is no significant difference (*p* > 0.05) between each group according to Tukey’s test for multiple comparisons. 24-SDC: 24-h-fermented Shuidouchi; 48-SDC: 48-h-fermented Shuidouchi; 72-SDC: 72-h-fermented Shuidouchi.

First black stool defecation time is an indicator of the intestinal patency and peristalsis. The first black stool defecation time for mice in the control group (140 ± 22 min) was the longest, but the time for mice in the normal group (63 ± 7 min) was shortest (Figure 4). Shuidouchi treatment could decrease the first black stool defecation time compared to that of the control group, and the first black stool defecation time of mice in the 72-SDC group (78 ± 6 min) was only slightly longer than that of mice in the normal group.

### 3.8. Gastrointestinal (GI) Transit in Constipated Mice

GI transit is also an indicator of the intestinal patency and peristalsis. The small-intestine lengths in different groups of mice were similar (Table 6), and the length of GI transit of mice in the normal group was the longest; with the induction of constipation, the length of GI transit was shorter (control group). Meanwhile, the activated carbon propulsive rate of mice in the normal group was the longest, and this rate was the shortest for mice in the control group. Shuidouchi could increase the length of GI transit and the activated carbon propulsive rate compared to the constipated mice (control group), and these effects of 72-SDC were superior to those of 48-SDC and 24-SDC.

### 3.9. Serum Ghrelin, ET-1, VIP, and AchE Levels in Mice

*Ghrelin*, ET-1, VIP and AchE levels are markers related to constipation in the blood [7]. The *ghrelin*, ET-1, VIP, and AchE levels of mice in the normal group were highest (Table 7); mice treated with 24-SDC, 48-SDC, and 72-SDC also showed higher *ghrelin*, ET-1, VIP, and AchE levels than those of control group mice. The *ghrelin*, ET-1, VIP, and AchE levels of mice in the 72-SDC group were only lower than those of mice in the control group. 

### 3.10. Morphological Observation of Small-Intestine Tissue

**Figure 5:**
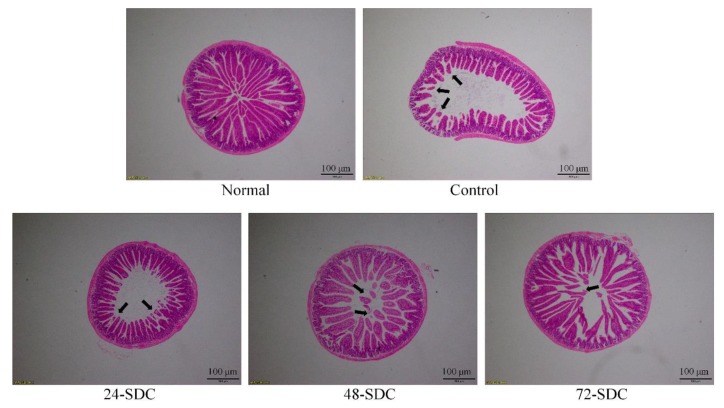
Morphological observation (40×) of small-intestine tissue in mice with diphenoxylate-induced constipation. 24-SDC: 24-h-fermented Shuidouchi; 48-SDC: 48-h-fermented Shuidouchi; 72-SDC: 72-h-fermented Shuidouchi.

Pathological observation is an important way to judge the degree of tissue damage intuitively [23]. As shown in Figure 5, the intestinal cells of normal mice were all distributed, and the villi of the small intestine were well arranged and well formed. The intestinal wall of the control group (diphenoxylate treatment) became thinner, and the villi of the small intestine broke up. At the same time, the cells of the small intestine were damaged. With the increase of fermentation time, 72-h-fermented Shuidouchi appeared to reduce these injuries.

### 3.11. c-Kit and SCF Expression of Small-Intestine Tissue in Mice

**Figure 6:**
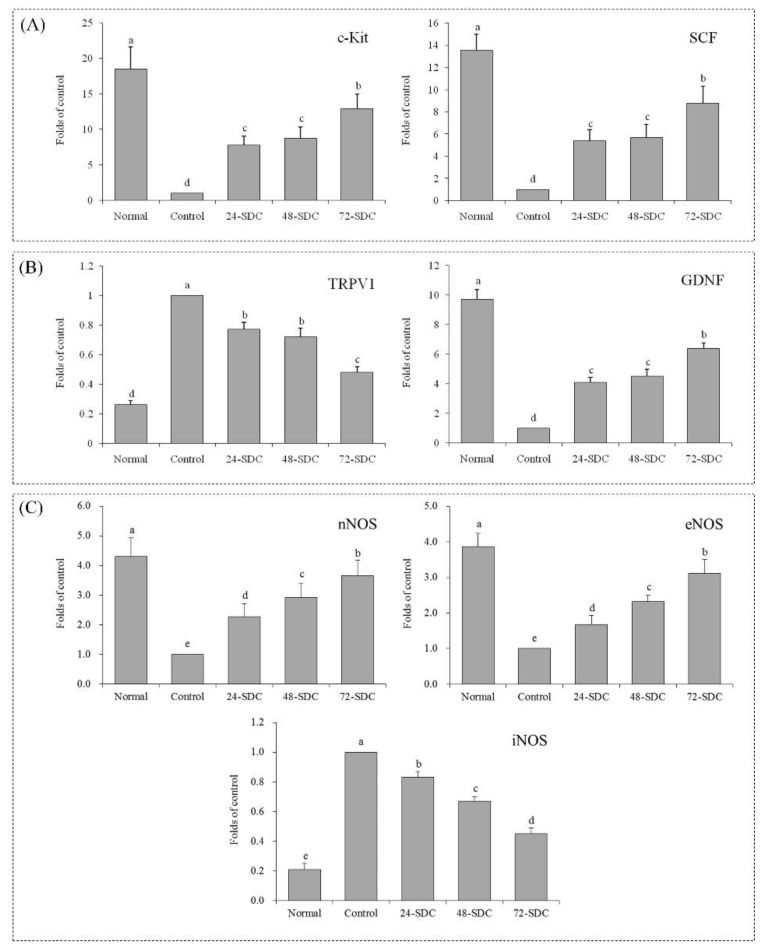
Messenger RNA (mRNA) expression levels of (**A**) *c-Kit* and stem-cell factor (*SCF*); (**B**): transient receptor potential cation channel subfamily V member 1 (*TRPV1*) and glial cell-derived neurotrophic factor (*GDNF*); (**C**) neuronal nitric oxide synthase (*nNOS*), endothelial nitric oxide synthase (*eNOS*), and; *iNOS*: inducible nitric oxide synthase (*iNOS*) in the small-intestine tissue of mice. Values presented are means ± standard deviation (*n* = 3). Different letters indicate significant differences (*p* < 0.05) between each group, and the same letters indicate that there is no significant difference (*p* > 0.05) between each group according to Tukey’s test for multiple comparisons. 24-SDC: 24-h-fermented Shuidouchi; 48-SDC: 48-h-fermented Shuidouchi; 72-SDC: 72-h-fermented Shuidouchi.

**Figure 7:**
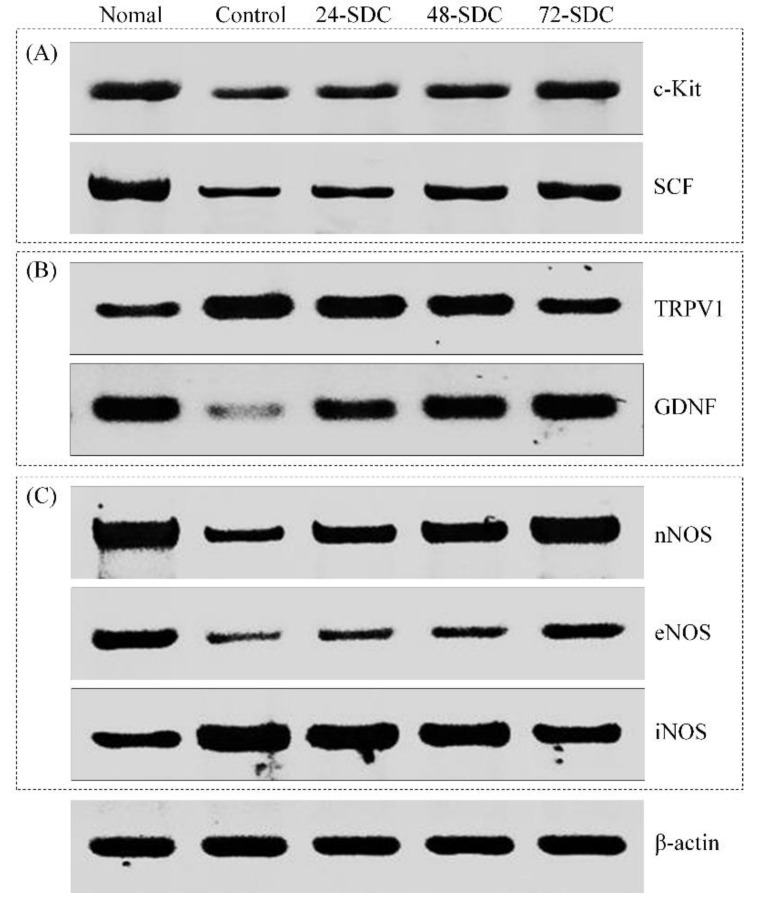
Protein expression levels of (**A**) *c-Kit* and *SCF*; (**B**) *TRPV1* and *GDNF*; (**C**) *nNOS*, *eNOS*, and *iNOS* in the small-intestine tissue of mice. 24-SDC: 24-h-fermented Shuidouchi; 48-SDC: 48-h-fermented Shuidouchi; 72-SDC: 72-h-fermented Shuidouchi.

Quantitative PCR and Western blot are important detection methods for molecular mechanisms. As shown in Figure 6A and Figure 7A, the *c-Kit* and *SCF* mRNA and protein expression levels of mice in the control group were the lowest, and these expression levels (18.47- and 13.56-fold mRNA expression compared to the control) of mice in the normal group were the highest. The *c-Kit* and *SCF* mRNA and protein expression levels of mice in the 24-SDC (7.79- and 5.36-fold mRNA expression compared to the control), 48-SDC (8.75- and 5.68-fold mRNA expression compared to the control), and 72-SDC (12.87- and 8.76-fold mRNA expression compared to the control) groups were also stronger than those of mice in the control group.

### 3.12. TRPV1 and GDNF Expression of Small-Intestine Tissue in Mice

As shown in Figure 6B and Figure 7B, the *TRPV1* mRNA and protein expression levels of mice in the control group were the strongest, and the *GDNF* mRNA and protein expression levels were the weakest. After treatment with Shuidouchi, the *TRPV1* expression levels were reduced, and the *GDNF* expression levels were raised; the *TRPV1* and *GDNF* expression levels of mice in the 72-SDC group (0.48- and 6.39-fold mRNA expression compared to the control) were closest to those in the mice in the normal group (0.26- and 9.72-fold mRNA expression compared to the control). 

### 3.13. nNOS, eNOS, and iNOS Expression of Small-Intestine Tissue in Mice

As shown in Figure 6C and Figure 7C, the *nNOS* and *eNOS* mRNA and protein expression levels in small-intestinal tissue of mice in the normal group were the strongest (4.31- and 3.87-fold mRNA expression compared to the control), but were the weakest in the control group. The *iNOS* mRNA and protein expression levels in mice of the control group were the strongest. After Shuidouchi treatment, the *nNOS* and *eNOS* mRNA and protein expression levels and the *iNOS* mRNA and protein expression levels decreased compared to those in the control group mice, and 72-SDC showed stronger increases (3.66-fold *nNOS* and 3.11-fold *eNOS* mRNA expression compared to the control) or decreases (0.45-fold *eNOS* mRNA expression compared to the control) than 24-SDC and 48-SDC.

### 3.14. Ghrelin Expression of Gastric Tissue in Mice

**Figure 8:**
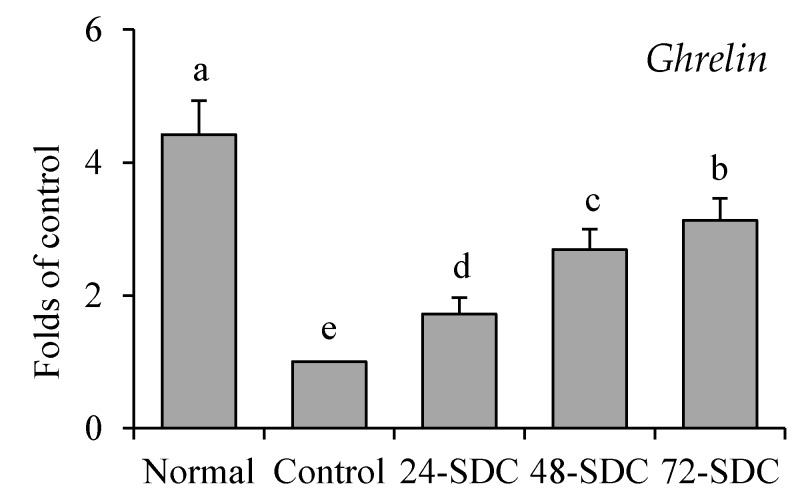
Messenger RNA level of *ghrelin* in gastric tissue of mice. Values presented are means ± standard deviation (*n* = 3). Different letters indicate significant differences (*p* < 0.05) between each group, and the same letters indicate that there is no significant difference (*p* > 0.05) between each group according to Tukey’s test for multiple comparisons. 24-SDC: 24-h-fermented Shuidouchi; 48-SDC: 48-h-fermented Shuidouchi; 72-SDC: 72-h-fermented Shuidouchi.

As shown in Figure 8, the *ghrelin* mRNA expression in gastric tissue of mice in the control group was the weakest, and this expression of mice in normal group was highest (4.42-fold mRNA expression compared to the control). Meanwhile the SDC-treated mice also showed higher *ghrelin* mRNA expression than control group mice, and the 72-SDC treated mice (3.13-fold mRNA expression compared to the control) had a higher *ghrelin* expression than 24-SDC (1.72-fold mRNA expression compared to the control) and 48-SDC (2.69-fold mRNA expression compared to the control) treated mice. 

### 3.15. TRPA1 Expression of Colon Tissue in Mice

**Figure 9:**
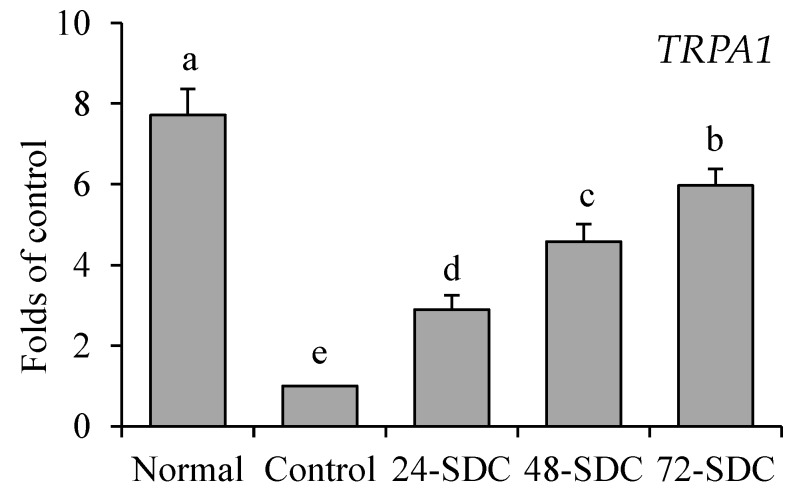
Messenger RNA level of transient receptor potential ankyrin 1 (*TRPA1*) in gastric tissue of mice. Values presented are means ± standard deviation (*n* = 3). Different letters indicate significant differences (*p* < 0.05) between each group, and the same letters indicate that there is no significant difference (*p* > 0.05) between each group according to Tukey’s test for multiple comparisons. 24-SDC: 24-h-fermented Shuidouchi; 48-SDC: 48-h-fermented Shuidouchi; 72-SDC: 72-h-fermented Shuidouchi.

As shown in Figure 9, the mice in the normal group (3.13-fold mRNA expression compared to the control) showed the strongest *TRPA1* mRNA expression in colon tissue of mice, and the 72-SDC (3.13-fold mRNA expression compared to the control), 48-SDC (3.13-fold mRNA expression compared to the control), and 24-SDC (3.13-fold mRNA expression compared to the control) treated mice also showed a stronger *TRPA1* expression than that of mice in the control group.

## 4. Discussion

In this study, through the constipation model, Shuidouchi could reduce the defecation time to excrete the first black stool and raise the GI transit compared to the control mice. Shuidouchi could also increase the serum levels of *ghrelin*, ET-1, VIP, and AchE in constipated mice. Moreover, Shuidouchi could upregulate the *c-Kit*, *SCF*, *GNDF*, *nNOS*, *eNOS*, *ghrelin*, and *TRPA1* expression and down-regulate the *TRPV1* and *iNOS* expression in constipated mice. Based on these data, Shuidouchi showed beneficial effects on the body state of constipated mice. With an increased time for fermentation of Shuidouchi, the number of microorganisms in the fermented Shuidouchi increased, and the acidity of the Shuidouchi increased. With the decrease in the pH value, the fermentation environment of the fermented bean was more powerful [1]. Beneficial microbes can inhibit the harmful microbes in the intestinal tract, improve the intestinal environment, promote intestinal peristalsis, and alleviate constipation. Beneficial microbes in fermented Shuidouchi could also play the above role; with the increase of fermentation time, the *B. subtilis* level in Shuidouchi also increased, and its effect of preventing constipation also increased [2,3]. Beneficial microorganism *Bacillus subtilis* could regulate somatostatin and was found to induce intestinal peristalsis to inhibit constipation [26]. *Bacillus subtilis* natto is also a kind of *Bacillus subtilis*, which might also have a functional effect on constipation. *Bacillus subtilis* natto is the main microorganism in Shuidouchi. *Bacillus subtilis* natto is the main source of intestinal function of Shuidouchi.

The in vitro small-intestine movement determination is a method for detecting intestinal irritation and intestinal viability. The increase in contraction frequency and intestinal muscle contractility could promote peristalsis of the small intestine, make the defecation smoother, and avoid constipation [18]. In vitro experiments proved that Shuidouchi has such effects. Shuidouchi, like natto from Japan, is rich in *Bacillus subtilis* natto. *Bacillus subtilis* natto, as a probiotic bacterium, was shown to have functional effects including intestinal protection [27]. In this study, the increase of intestinal microorganisms in mice after eating Shuidouchi was due to the introduction of *Bacillus subtilis* natto after eating Shuidouchi. *Bacillus subtilis* natto of Shuidouchi might play an important role in the intestine of mice to inhibit constipation.

Genistein and raffinose are active ingredients in Shuidouchi, which play an important role in the physiological activity of Shuidouchi [3]. Raffinose is an excellent *Bifidobacterium* multiplication factor, and has a strong intestinal protective effect [28]. Raffinose is an important component of soybean oligosaccharides. In the process of fermentation, the effect of microbes can greatly improve the content of soybean oligosaccharides in fermented soybean products and can also help to repair intestinal damage [29]. Probiotics can also produce gas when they decompose raffinose; however, because raffinose promotes the proliferation of probiotics, the beneficial effect is greater than the disadvantage of gas production; thus, raffinose is more conducive to alleviating constipation [30]. Soybean isoflavone is an important physiologically active substance in soybeans. The fermentation process of soybean fermented food can transform soybean isoflavone into free soybean isoflavone, which can be easily absorbed by the human body [31]. Genistein is an important free soybean isoflavone in fermented soybean food. It also has a good protective effect on the intestinal tract, and the combined effects of beneficial microorganisms are also beneficial for the peristalsis of the intestines and relief of constipation [10]. The 72-SDC group showed higher raffinose and genistein contents than 24-SDC and 48-SDC, and the high contents of raffinose and genistein were good for protecting the body, whereby they could help improve intestinal function.

Patients with constipation have difficulty in defecation, and the frequency of defecation and the level of probiotics are lower than that in normal people. The mouse constipation model simulates constipation in human body. Under the state of difficult defecation, the defecation time of black stool produced by activated carbon water is a standard to measure the degree of constipation [32]. The results show that the black stool defecation time of mice eating 72-SDC feed was significantly lower than that of mice eating 24-SDC and 48-SDC feed (*p* < 0.05). Thus, 72-h fermentation of Shuidouchi is more advantageous to promote intestinal health and defecation. The most obvious manifestations of constipation are difficult defecation, smaller amount of excrement, and dry particles due to low water content [33]. Shuidouchi can improve defecation, and the effects of 72-SDC were better than those of 24-SDC and 48-SDC.

Propulsion distance and rate of activated carbon in the small intestine are important indicators to measure small-intestine function and help judge the degree of constipation in mice. Longer distances and higher propulsion rates indicate a lower degree of constipation [34]. The results show that Shuidouchi caused black stool to spend less time in the intestinal tract and pass through the small intestine faster. Compared to 24-SDC and 48-SDC, 72-SDC had a better effect, whereby 72-SDC could enable the activated carbon to pass through the small intestine faster.

Endothelin can maintain vascular tension in the normal cardiovascular system and avoid other diseases caused by constipation. ET-1 is an important factor that regulates cardiovascular function; it can promote normal contraction and diastole of blood vessels and relieve abnormal contractions caused by constipation [35,36]. VIP can relax smooth muscles, have vasodilation effects, increase small-intestinal secretions, and stimulate intestinal peristalsis; thus, a decrease in VIP secretion can directly lead to constipation [37]. AchE can adjust intestinal contraction and promote secretion of mucus. By enhancing intestinal contraction and intestinal mucous secretion, feces can be excreted more easily, thus avoiding constipation [38]. In this study, Shuidouchi had significant effects on ET-1, VIP, and AchE in mice, and it can be concluded that Shuidouchi can relieve constipation by regulating the levels of ET-1, VIP, and AchE.

Cajal interstitial cells (ICCs) are cells that play an important role between the enteric nervous system (ENS) and smooth muscles. ICCs can regulate the intestinal nerve signals of smooth-muscle cells, and clinical studies showed that, under constipation conditions, ICC levels in the body drop. *c-Kit* is the specific marker of ICCs; thus, it can be used as an important standard to observe the level of ICC [39]. ICCs only exist under a certain concentration of *SCF*. Blocking the combination between *c-Kit* and *SCF* can reduce ICC levels, and a decrease in *SCF* concentration will lead to a decrease in *c-Kit* concentration; thus, high expression of *SCF* plays a key role in the survival of ICCs in the small-intestine tissue [40]. Shuidouchi can effectively upregulate the expression of *c-Kit* and *SCF* in the small-intestine tissue of mice, which may help relieve constipation by raising the level of ICCs in the body of mice.

The release of neurotransmitters by stimulating *TRPV1* can cause intestinal motility disorder, affect defecation, and form constipation. Because intestinal injury can cause intestinal disturbance, the expression of *TRPV1* in the small intestine will become stronger under constipation [41]. *GDNF* can control the growth and development of nerve cells and protect and repair damaged nerve fibers, which can help repair damaged intestines and prevent constipation [32]. Endogenous NO widely exists in the gastrointestinal tract, and *NOS* is the key enzyme that produces NO. Large quantities of NO can cause gastrointestinal dysmotility; hence, controlling the expression of *NOS* can effectively reduce the content of NO, thus relieving constipation [42]. The results of this study also confirmed that Shuidouchi enhances intestinal activity by upregulating the expression of *GDNF* and downregulating the expression of *TRPV1* in the small-intestine tissue of mice, and protecting the function of the small intestine.

*NOS* can catalyze the synthesis of nitric oxide (NO), and NO is one of the main inhibitory neurotransmitters in the small-intestinal plexus. NO plays an important role in gastrointestinal dynamic regulation. Under normal physiological conditions, *iNOS* is not active; however, under stimulation by tissue injury, its activity can be detected in vivo [43]. On the other hand, *nNOS* participates in a series of physiological and pathological processes, including nerve transmission, neurotoxicity, and skeletal muscle contraction [44]. Finally, *eNOS* is an important regulator of cardiovascular homeostasis. It can regulate the diameter of blood vessels and maintain the anti-proliferative and anti-apoptotic environment of the vascular system [45]. Both can regulate small-intestinal nerves and intestinal muscle groups, which can help relieve constipation. Therefore, effectively reducing the expression of *TRPV1* and *NOS*, and strengthening the expression of *GDNF* in the small intestine is an important way of controlling and alleviating constipation, and 72-SDC could better upregulate the expression of *GDNF* and downregulate the expression of *TRPV1* and *NOS* in the small-intestine tissue of mice than Shuidouchi with other fermentation times; thus, it can effectively prevent constipation.

*Ghrelin* can connect the gastrointestinal tract and the central nervous system through paracrine, autocrine, and endocrine functions, and regulate the digestive system function. *Ghrelin* plays a central and peripheral regulatory role in stimulating appetite and participating in gastric acid secretion through the vagus nerve. It also accelerates normal gastric emptying [46]. *TRPA1* is widely distributed in the gastrointestinal tract, which is involved in gastrointestinal signal transduction and intestinal motility regulation. *TRPA1* can significantly enhance colonic and gastric contraction and enhance colonic and gastric transport function, especially in a concentration-dependent manner [47]. Similarly, the *ghrelin* expression in gastric tissues and *TRPA1* expression in intestinal tissues were enhanced after SDC improved constipation. Thus, SDC could effectively enhance the expression of *ghrelin* and *TRPA1* in mice gastric tissues to alleviate constipation.

Shuidouchi contains bioactive substances beneficial to the gastrointestinal tract, and also contains a large number of beneficial bacteria. The active ingredients of Shuidouchi could protect the small-intestinal nerve and stimulate small-intestinal peristalsis in mice. The beneficial microorganisms in Shuidouchi could improve the micro-ecological environment of the small intestine, keep the small intestine healthy, and promote defecation. The combined action of these substances makes Shuidouchi inhibit constipation in mice. All these functional ingredients show that Shuidouchi has a good effect on health. If these functional ingredients come into play, Shuidouchi can improve body discomfort. In this study, animal experiments were conducted to verify the constipation inhibitory effect of Shuidouchi. In the future, further human clinical studies are needed to verify the findings of this study. At the same time, according to the active ingredient analysis of Shuidouchi, more in-depth mechanism research is also necessary.

## 5. Conclusions

An animal model was used to determine the constipation inhibition effects of Shuidouchi in this study. The results showed that 72-SDC-treated mice had shorter first black stool defecation times than 24-SDC- or 48-SDC-treated mice and control group mice. Meanwhile, 72-SDC-treated mice had a higher activated carbon propulsive rate than other groups of mice, except for the normal group mice. Moreover, 72-SDC could also raise *Ghrelin*, ET-1, VIP and AchE serum levels in constipation mice. Additionally, 72-SDC increased *c-Kit*, *SCF*, *GNDF*, *nNOS*, and *eNOS* mRNA and protein expression levels, and decreased *TRPV1* and *iNOS* expression levels compared to those of the control group. It was found that 72-SDC inhibited constipation more effectively than 24-SDC and 48-SDC. Shuidouchi could promote intestinal motility, strengthen intestinal peristalsis of constipated mice, and improve the stool of constipated mice. From these results, Shuidouchi has a good preventative effect on experimental constipation in mice, and 72-h fermentation had the best effects. 

## Figures and Tables

**Table 1:** Sequences of primers used in this study.

Gene Name	Sequence
*c-Kit*	Forward: 5′-CATAGCCCAGGTAAAGCACAAT-3′
	Reverse: 5′-GAACACTCCAGAATCGTCAACTC-3′
*SCF*	Forward: 5′-TCAGGGACTACGCTGCGAAAG-3′
	Reverse: 5′-AAGAGCTGGCAGACCGACTCA-3′
*TRPV1*	Forward: 5′-CCGGCTTTTTGGGAAGGGT-3′
	Reverse: 5′-GAGACAGGTAGGTCCATCCAC-3′
*GDNF*	Forward: 5′-GGGGTATGGAGAAGTTGGCTAG-3′
	Reverse: 5′-CTATGAGAATGCTGCCGAAAA-3′
*nNOS*	Forward: 5′-GAGAGGATTCTGAAGATGAGG-3′
	Reverse: 5′-TTGCTAATGAGGGAGTTGTTC-3′
*eNOS*	Forward: 5′-TGTTTGTCTGCGGCGATGT-3′
	Reverse: 5′-GGGTGCGTATGCGGCTTGTC-3′
*iNOS*	Forward: 5′-CATTGGAAGTGAAGCGTTTCG-3′
	Reverse: 5′-CACAGAACTGAGGGTACA-3′
*Ghrelin*	Forward: 5′-TTGAGCCCAGAGCACCAGAAA-3′
	Reverse: 5′-AGTTGCAGAGGAGGCAGAAGC-3′
*TRPA1*	Forward: 5′-AATCTCTGTCCTCTGCATCACG-3′
	Reverse: 5′-ACAATGCAGTGGGGTATTTCC-3′
*GAPDH*	Forward: 5′-TGCACCACCAACTGCTTAG-3′
	Reverse: 5′-GATGCAGGGATGATGTTC-3′

*SCF*: stem-cell factor; *TRPV1*: transient receptor potential cation channel subfamily V member 1; *GDNF*: glial cell-derived neurotrophic factor; *nNOS*: neuronal nitric oxide synthase; *eNOS*: endothelial nitric oxide synthase; *iNOS*: inducible nitric oxide synthase; *TRPA1*: transient receptor potential ankyrin 1; *GAPDH*: glyceraldehyde-3-phosphate dehydrogenase.

**Table 2:** The pH, acidity, and total viable counts of Shuidouchi (SDC) at different fermentation times.

Groups	24-SDC	48-SDC	72-SDC
pH	7.12 ± 0.06 ^c^	6.71 ± 0.09 ^b^	6.18 ± 0.05 ^a^
Acidity (%)	1.21 ± 0.08 ^c^	1.48 ± 0.05 ^b^	1.63 ± 0.05 ^a^
Total viable counts (×10^9^ colony-forming units (CFU)/g)	1.80 ± 0.22 ^c^	2.35 ± 0.18 ^b^	3.82 ± 0.25 ^a^

Values presented are means ± standard deviation (*n* = 3). Different letters indicate significant differences (*p* < 0.05) between each group, and the same letters indicate that there is no significant difference (*p* > 0.05) between each group according to Tukey’s test for multiple comparisons. 24-SDC: 24-h-fermented Shuidouchi; 48-SDC: 48-h-fermented Shuidouchi; 72-SDC: 72-h-fermented Shuidouchi.

**Table 3:** Physiological and biochemical identification results of the most important strain isolated from Shuidouchi.

Index	Positive (+)/Negative (−)	Index	Positive (+)/Negative (−)
Glucose	+	Catalase	+
Sucrose	−	V-P determination	+
d-Galactose	−	Methyl red	+
Lactose	+	Starch	+
Maltose	+	Nitrate reduction	+
d-Xylose	+	Nitrite reduction	+
d-Fructose	−	Lipoidase (Tween 60)	+
Cellobiose	−	Isinglass	+
Arabinose	+	Cow milk	+
Raffinose	+	Indole	−
Mannite	+	Citrate	+
Xylitol	−	Acetic acid oxidation	+
Phaseomannite	−	Lecithinase	−

**Table 4:** In vitro small-intestine movement effects of raffinose, genistein, and SDC.

Groups	Raffinose	Genistein	24-SDC	48-SDC	72-SDC
Contraction frequency (frequency/min)	125 ± 14 ^ab^	147 ± 12 ^a^	88 ± 7 ^c^	95 ± 8 ^c^	116 ± 12 ^ab^
Intestinal muscle contractility (g)	4.1 ± 0.2 ^a^	4.2 ± 0.2^a^	3.5 ± 0.2 ^b^	3.6 ± 0.1 ^b^	4.0 ± 0.2 ^a^

Values presented are means ± standard deviation (*n* = 3). Different letters indicate significant differences (*p* < 0.05) between each group, and the same letters indicate that there is no significant difference (*p* > 0.05) between each group according to Tukey’s test for multiple comparisons. 24-SDC: 24-h-fermented Shuidouchi; 48-SDC: 48-h-fermented Shuidouchi; 72-SDC: 72-h-fermented Shuidouchi.

**Table 5:** Stool status of Shuidouchi-treated mice at the last day of the experiment.

Groups	Normal	Control	24-SDC	48-SDC	72-SDC
Stool weight (g)	0.92 ± 0.03 ^a^	0.39 ± 0.05 ^d^	0.67 ± 0.05 ^c^	0.73 ± 0.05 ^bc^	0.78 ± 0.04 ^b^
Particle counts of stool	43 ± 3 ^a^	15 ± 2 ^d^	29 ± 3 ^c^	31 ± 3 ^c^	38 ± 4 ^b^
Water content of stool (%)	47 ± 3 ^a^	18 ± 3 ^c^	38 ± 4 ^b^	39 ± 4 ^b^	42 ± 4 ^b^
Total viable counts of stool (×10^9^ CFU/g)	2.35 ± 0.21 ^a^	1.65 ± 0.26 ^d^	1.84 ± 0.16 ^d^	1.91 ± 0.12 ^c^^d^	2.24 ± 0.15 ^b^

Values presented are means ± standard deviation (*n* = 10). Different letters indicate significant differences (*p* < 0.05) between each group, and the same letters indicate that there is no significant difference (*p* > 0.05) between each group according to Tukey’s test for multiple comparisons. 24-SDC: 24-h-fermented Shuidouchi; 48-SDC: 48-h-fermented Shuidouchi; 72-SDC: 72-h-fermented Shuidouchi.

**Table 6:** Effects of Shuidouchi on gastrointestinal (GI) transit in 30 min in constipated mice.

Groups	Normal	Control	24-SDC	48-SDC	72-SDC
Length of small intestine (cm)	49.7 ± 2.6 ^a^	49.6 ± 2.2 ^a^	50.2 ± 2.4 ^a^	49.9 ± 2.2 ^a^	50.0 ± 2.5 ^a^
Length of GI transit (cm)	48.8 ± 2.1 ^a^	12.7 ± 2.3 ^d^	28.7 ± 2.1 ^c^	31.2 ± 2.2 ^c^	39.5 ± 2.3 ^b^
Activated carbon propulsive rate (%)	98.2 ± 1.5 ^a^	25.6 ± 2.6 ^e^	57.2 ± 2.4 ^d^	62.5 ± 2.5 ^c^	79.0 ± 2.6 ^b^

Values presented are means ± standard deviation (*n* = 5). Different letters indicate significant differences (*p* < 0.05) between each group, and the same letters indicate that there is no significant difference (*p* > 0.05) between each group according to Tukey’s test for multiple comparisons. 24-SDC: 24-h-fermented Shuidouchi; 48-SDC: 48-h-fermented Shuidouchi; 72-SDC: 72-h-fermented Shuidouchi.

**Table 7:** *Ghrelin*, endothelin-1 (ET-1), vasoactive intestinal peptide (VIP), and acetylcholinesterase (AchE) serum levels in mice with diphenoxylate-induced constipation.

Groups	Normal	Control	24-SDC	48-SDC	72-SDC
*Ghrelin* (ng/mL)	62.6 ± 3.5 ^a^	34.5 ± 3.3 ^e^	40.1 ± 1.8 ^d^	44.1 ± 1.7 ^c^	50.3 ± 2.3 ^b^
ET-1 (pg/mL)	19.1 ± 1.7 ^a^	6.1 ± 0.4 ^d^	10.6 ± 1.0 ^c^	11.8 ± 1.1 ^c^	14.7 ± 0.9 ^b^
VIP (pg/mL)	80.3 ± 5.1 ^a^	33.3 ± 2.6 ^e^	51.2 ± 2.1 ^d^	57.3 ± 2.2 ^c^	68.7 ± 1.9 ^b^
AchE (pg/mL)	51.2 ± 2.2 ^a^	15.5 ± 1.7 ^e^	30.2 ± 1.5 ^d^	35.6 ± 1.8 ^c^	43.6 ± 1.3 ^b^

Values presented are means ± standard deviation (*n* = 10). Different letters indicate significant differences (*p* < 0.05) between each group, and the same letters indicate that there is no significant difference (*p* > 0.05) between each group according to Tukey’s test for multiple comparisons. 24-SDC: 24-h-fermented Shuidouchi; 48-SDC: 48-h-fermented Shuidouchi; 72-SDC: 72-h-fermented Shuidouchi.
